# How to Sleep Well

**Published:** 1881-12

**Authors:** 


### HOW TO SLEEP WELL.

NO healthful sleep comes except that which follows voluntary or involuntary action of the muscles of the body. Pedestrians fall into sound, deep sleep as soon as put to bed, at the appointed time for rest. This is the sleep from voluntary muscular exercise. A person in good health sits around the house all day ; an invalid may all day sit, and lounge, and lie down from morning until night without sleeping ; and both the healthy man and the invalid, in the course of the evening, will become sleepy, and fall into sound repose, the result of the weariness which involuntary motion brings about ; for the various organs of the body, the heart, the liver, the stomach, the eyelids, work steadily every day. The intestines are as ceaseless in their motion as the waves of the ocean ; as these latter are always dashing toward the shore, so is the great visceral machinery working, working, working, pushing the wastes of the body downward and outward from the first breath of existence to the last gasp of life.

There is not a movement of the system, voluntary or involuntary, external or internal, which does not require power to cause it. When that power is to a certain extent exhausted, instinct brings on the sensation of sleepiness, which is the result of exhausted power, intended by nature to secure that cessation from activity which gives time for recuperation, very much as a man who runs for a while stops and rests, so as to get stronger to run again.

We get up in the morning with a certain amount of reserved or accumulated strength ; in the course of the day that strength becomes expended to the point necessary for the commencement of a new supply, which supply comes from rest, the rest from sleep.

Opium, narcotics, all forms of anodynes, cause sleep artificially, by compelling rest. A horse may be tied so that he cannot move ; he is compelled to be at rest; it is not the rest of tiredness, hence it is unnatural. Anodynes, in a sense, tie a man down; they take away his power of motion, they compel a rest, but it is not the rest which is a result of used-up strength, hence it is an artificial rest, causing artificial sleep, not natural; and sleep which is not natural cannot be healthful; hence the truth of the first utterance of this chapter: healthful sleep comes from the expenditure of the strength of the body in various forms of exercise.

